# Editorial: Multi-Actor Collaboration in Healthcare to Address the Emerging Health Needs of an Aging Population

**Published:** 2019-01-06

**Authors:** M Illario, E Coscioni, V De Luca, M Cataldi, A Postiglione, G Iaccarino

**Affiliations:** 1 Campania Region Division for Health Innovation, and Department of Public Health, Federico II University and Hospital, Naples, Italy; 2 Department of Heart Surgery, San Giovanni di Dio e Ruggi d’Aragona Hospital, Salerno, Italy; 3 Research and Development Unit, Federico II University Hospital, Naples, Italy; 4 Department of Neurosciences, Reproductive Sciences and Odontostomatology, University of Naples Federico II, Naples, Italy; 5 General Directorate for Health Protection and the Coordination of Regional Health System, Campania Region, Naples, Italy; 6 Department of Advanced Biomedical Sciences, University of Naples Federico II, Naples, Italy

## I. EDITORIAL

This special issue is nested in the A3 Action Group of the European Innovation Partnership on Active and Healthy Ageing (EIP on AHA), focusing on lifecourse health promotion and prevention of age-related frailty and disease. The ambition is to share knowledge underpinning the transfer of innovations among stakeholders, and facilitate adoption by Regional Health Systems, in different EU contexts.

Health and societal challenges require the ever faster availability of new approaches to prevention, diagnostics and care, which pose important sustainability and equity issues to regions. The growing economic burden mandates the adoption of new forms of service provision, that improve sustainability and allow to face the current demographic challenges. The ageing trend in European populations is indeed paralleled by a rise in multimorbidity and comorbidity, and a clustering of chronic diseases often correlated with each other.

Coping with multimorbid patients raises issues of clinical management, as it is necessary to follow multiple indications and guidelines (for each individual disease), while embedding lifestyles measures that can significantly influence outcomes. The consequence are therapeutic inconsistencies, redundant diagnostic pathways, while struggling with adherence, and increasing in healthcare costs. Hence the urge to define new care pathways, which are able to take care of the patient, ensuring both the continuity of hospital-territory care, and the integration of social and health interventions.

The wide-scale implementation of validated innovative approaches is essential to ensure the quality of services to future generations of citizens in an acceptable range of sustainability, avoiding horizontal cuts that would increase inequalities and worsen health outcomes in the long term. It is therefore essential to set up models that are sustainable in an overall health innovation process, where ongoing structural reforms increase the effectiveness and resilience of our health systems. In this framework, disruptive innovations can address sustainably and appropriately the health needs of citizens. Often, the absence of a coordinated approach to social and health reforms is associated with the inadequate participation of citizens, patients, formal and informal caregivers in their planning, set-up and evaluation. Isolated approaches undermine the evolution towards an integrated and high quality health system, that it is also sustainable, efficient and fair. These difficulties are greater in the older adults population, which has multiple health needs, and minor access to services, for reasons often unrelated to the diseases (e.g. IT gaps, mobility problems, residence in rural areas, etc.).

One way to achieve these objectives are ICT enabled integrated health and social care services (ICT-IC). The development of digital health tools allows to find new answers to traditional problems in patient management, as well as to improve the services by strengthening the collaboration between the different professionals and caregivers involved inside and outside the health system. Furthermore, digital services improve accessibility, thus ensuring equity for example by making qualified services available also in remote, underserved areas.

The advantage of the new organizational models based on ICT-supported integrated care is the potential rationalization of social and health processes that impacts on the containment of health expenditure, and also reduces the social cost of diseases. If appropriately used, ICT based integrated services can contribute to a transformation of the health sector and a substantial change in the business models that underpin it.

The technological and digital innovations, if designed in a targeted manner and implemented on the basis of cost-effective criteria, provide tools capable of supporting the modernization of social and health systems, and their adaptation to challenges such as the aging of the population, especially in a context of sharing resources and skills.

This paradigm shift has the ambition to put the citizen at the center, while responding appropriately, sustainably and innovatively to individual and collective health needs. The adoption of advanced technologies for diagnostics and therapy, and the digitization of services and care represent an opportunity to be seized to trigger a virtuous circuit that connects needs, innovation and investments, through the adoption of transparent and responsible procedures. The multi-actor collaboration to the provision of services and engagement of citizens through networking models allows multidisciplinary management of innovative tools, while supporting the training of operators, the empowerment of citizens and the monitoring of health outcomes, also through rationalization and centralized management of resources. By doing so, clinical problems can be managed and solved. [1]

The objective of this special issue is to facilitate the dissemination of innovative approaches potentially able to impact older adults quality of life. This volume contains 16 thoroughly refereed contributions concerning a wide range of topics in active and healthy ageing and digital transformation of health and care.

Coherently with the scope of the special issue, Hendry et al. explain that frailty requires concerted integrated approaches to prevent functional decline. Authors provide evidence that few models of integrated care were specifically designed to prevent and tackle frailty in the community and at the interface between primary and secondary (hospital) care. Their experience strengthens current evidence, supporting the case for a more holistic and salutogenic response to frailty [2].

Comprehensive assessment and multidimensional interventions hence need to be tailored to modifiable physical, psychological, cognitive and social factors. To this purpose, the interregional MIND&GAIT project preliminary results demonstrate that social facilities such residential structures and day-centres are currently adopting such approach, and describe how they seek integrated, structured, adapted, creative, dynamic and economic strategies to prevent frailty, improving cognition and gait ability by using assistive products. [3]

The independent components of physical frailty that most influence disability indicators in institutionalized older women, that Furtado et al. analysed, help to better understand the independent relationship of each with disability outcomes and assist to design a co-adjuvant treatment to reverse physical frailty. [4]

It is commonly accepted that frailty and dementia-related cognitive decline are strongly associated. Kleisiaris et al. assessed the degree of this association especially in homebound elders with disabilities. The study aims to investigate the association of frailty on cognitive function in older adults receiving homecare. [5]

The paradigmatic shift to more resilient health systems requires a proactive approach: this is why the protocol of the ModulEn study aims to develop and validate a predictive model for frailty. The approach allows proactive and continuous monitoring of circadian health, physical activity, and sleep and eating habits. In the view of Apóstolo et al., the implications of this study’ findings for clinical practice include the possibility to develop and validate tools for timely prevention of frailty progress. [6] In the same line of proactive and preventive approach, Cataldi et al. developed and tested an innovative physical training method in older adults that embeds the gym program into everyday life in the most conservative way possible. The social role of parishes in Southern Italy is testified by the large number of elederlies that attend their daily activities. Physical training was included among those activities and delivered by physical therapists with ICT enabled physical training platform “CoCo”. The training program designed to minimally impact on life habits of older people is effective in improving fitness parameters in sedentary elederly subjects, noncompliant to other physical exercise programs [7].

Complex health needs require multiprofessional knowledge, that is hard to manage in the daily routine of healthcare professionals. The PITeS TIiSS project develops decision support tools to improve quality of life of multimorbid and polymedicated patients, while facilitating health care service management. Importantly, an ontology has been developed as a tool on adherence. The domain of this ontology is mainly focused on medication adherence and measurement methods, while gathering the necessary knowledge about the domain.[8]

The caregivers of home palliative care present needs and changes in the lifestyles. Their assessment helps to improve home services and relations with health system. Zavagli et al. have completed a battery of self-report questionnaires, including the Caregiving Tasks Consequences and Needs Questionnaire (CaTCoN), that measures caregivers’ experiences (the extent of cancer caregiving tasks and consequences) and the caregivers’ needs, mainly concerning the interaction with the health care professionals.[9]

Innovative approaches to cardiovascular related organ damage are also pivotal in a context of increasing chronic diseases. This is why one of the papers evaluates the short- and medium-term outcomes of Carotid artery stenting (CAS) performed with a single type of closed-cell stent design and distal filter protection. The study compares the procedure against Carotid artery endarterectomy (CEA) based upon clinically relevant endpoints (overall survival rate, stroke free survival rate and restenosis free survival rate) that affect the daily quality of life of chronic patients. [10]

Scaling up the adoption of innovative approaches is facilitated by collaborations and exchange of good practices along many different, inter-twined directories. This is why, in order to ensure that we are able to accelerate the digital transformation of health and care, a networking approach is adopted.[11]

RAPid COmmunity COGnitive screening Programme (RAPCOG) twinning project validated translated versions of the Quick Mild Cognitive Impairment (Q*mci*) screen that could be adapted quickly for use with future eHealth screening and assessment programmes. [12] Once identified, though, tailored interventions are needed to promote active ageing and independent living, and to prevent loss of functionality and autonomy. To achieve this goal the ECOG project promotes active citizenship in old age through empowerment for autonomy and self-care along three organizing axes: evidence synthesis, the establishment of good practice and transfer of good practice to different healthcare contexts [13].

Twinning ARIA project promotes adoption of mobile phone Apps for the management of allergic respiratory disease, through Mobile Airways Sentinel networK (MASK), the Phase 3 of the ARIA initiative, based on the freely available Allergy Diary App. [14]

SHAFE is a thematic network aimed at facilitating the creation of healthy and friendly environments for all ages through the use of new technologies. It is intended to highlight the importance of People and Places in the creation of digital solutions for eHealth and mHealth, with better quality, but still accessible to all. [15]

“Age-friendly” tourism is an example of an innovative services that strives to meet the health needs of the entire “traveling” population, with an integrated and cross-sector approach that involves various organizations operating in sectors such as ageing, healthcare, accessibility and transport. [16]

The Reference Site Collaborative Network (RSCN) brings together the EIP on AHA Reference Sites awarded by the European Commission, and Candidate Reference Sites into a single forum. The overarching goals are to promote cooperation, share and transfer good practice and solutions in the development and scaling up of health and care strategies, policies and service delivery models, while at the same time supporting the action groups in their work. Campania Reference Site is an active partner of the RSCN, and provides the opportunity to connect loco-regional stakeholders that can positively contribute to the digital transformation and integration of social and health care services (universities, healthcare providers, social services, local communities and municipalities, citizens), with international organizations, in order to adopt and scale up innovative solutions and approaches. [17]

The ageing trend of the EU population is a success story of our health systems, and is an asset worth our investments. Strong collaborative approaches that tare aligned in a shared vision will ensure they further develop in the future, and provide guidance wherever needed world-wide. “Blueprint on Digital Transformation of Health and Care for the Ageing Society” is guiding the shift towards ICT enabled patient-centered care. European Commission created a tool to explore the digital services landscape and find services that address the needs of the personas developed for this tool, that represent the needs of the populations. Lazic et al. explores the digital services ecosystem in primary care in Zagreb from the services availability and accessibility perspective, using the personas needs tool.[18]
